# Effects of Different Non-*Saccharomyces* Strains in Simultaneous and Sequential Co-Fermentations with *Saccharomyces cerevisiae* on the Quality Characteristics of Kiwi Wine

**DOI:** 10.3390/foods13162599

**Published:** 2024-08-20

**Authors:** Jie Zhang, Pengyan Li, Peiyao Zhang, Tieru Wang, Jianrui Sun, Libo Wang, Zhouya Bai, Jiangfeng Yuan, Lina Zhao, Shaobin Gu

**Affiliations:** 1 College of Food and Bioengineering, Henan University of Science and Technology, Luoyang 471023, China; superlipengyan@126.com (P.L.); 15939735097@163.com (P.Z.); dasheng@haust.edu.cn (J.S.); lbwang0728@163.com (L.W.); spbaizhouya@163.com (Z.B.); jiangfengyuan@163.com (J.Y.); zhaolina@haust.edu.cn (L.Z.); shaobingu@haust.edu.cn (S.G.); 2 College of Food Science and Engineering, Northwest A & F University, Xianyang 712100, China; 2012015240@nwsuaf.edu.cn

**Keywords:** non-*Saccharomyces* yeasts, mixed fermentation, kiwi wine, phenolic profiles, antioxidant ability, organic acids

## Abstract

With the increasing awareness of health, more people have shown a preference for low-alcohol beverages. Seeking various methods to improve the quality of kiwi wine is now a major research interest in the wine industry. In this study, kiwi wine was fermented by *Saccharomyces cerevisiae* and different non-*Saccharomyces* strains (*Torulaspora delbrueckii*, *Kluyveromyces thermotolerans*, *Pichia fermentans*) in three methods (pure fermentation, simultaneous, and sequential co-fermentation). The physicochemical characteristics, color parameters, phenolic profiles, total phenolic content (TPC), antioxidant activities, organic acids, and taste sense of the different wines were evaluated to determine the effects of different yeasts and fermentation methods on the quality of the kiwi wine. Results indicated that co-fermentation reduced the contents of alcohol while enhancing the lightness of the kiwi wine. The TPC of sequential co-fermentation with *K. thermotolerans*/*S. cerevisiae* was significantly higher than that of their simultaneous co-fermentation. Compared to *K. thermotolerans*/*S. cerevisiae*, the antioxidant activities were increased by co-fermentation of *T. delbrueckii*/*S. cerevisiae* and *P. fermentans*/*S. cerevisiae*. Principal component analysis showed that kiwi wines fermented by different yeasts and inoculation methods could be separated and grouped. Correlation analysis presented positive correlations of phenolic composition, antioxidant activities, and color intensity. This study provided theoretical guidance for co-fermentation of non-*Saccharomyces*/*S. cerevisiae* and accelerated the industrialization process of kiwi wine.

## 1. Introduction

Kiwifruit (*Actinidia deliciosa*) contains abundant amounts of bioactive ingredients including vitamin C, chlorophylls, and carotenoids, which are beneficial to human health [1]. As one of the ultra-processed products of kiwifruit, kiwi wine has broad market prospects, and developing high quality kiwi wine is the key to enhancing its product value [2]. There are many factors that affect the quality of kiwi wine, such as its antioxidant properties and color [3]. Scavenging models of DPPH radicals, hydroxyl radicals, and superoxide anion radicals are widely used to assess the antioxidant activity of kiwi wine [4,5]. The antioxidant performance of kiwi wine is also closely associated with phenolic compounds. Phenolic compounds, especially polyphenols, have been proven to reduce the risks of certain chronic disease and prevent the development of cardiovascular diseases and diabetes [6,7]. Therefore, phenolic compounds play an important role in evaluating the product quality of kiwi wine and enhancing its market competitiveness [8]. With regard to color, it is considered as one of the parameters directly determining the quality of fruit wine and further influences consumer acceptance [9]. The color change is linked to the changes of some bioactive ingredients in kiwi wine. For example, anthocyanin-flavanol condensation reactions and flavanols-involved oxidative browning reactions have been demonstrated to occur during wine aging, which give rise to important changes in the color of wine [10]. At present, the correlation between the color change in kiwi wine and its bioactive ingredients has become a hot spot of research.

As the main microbe in the alcohol fermentation process, yeast plays an important role, affecting kiwi wine’s bioactive components and sensory properties. The natural alcohol fermentation of wine usually starts by low-alcohol-tolerant apiculate yeasts, which dominate the first fermentation stage and are later replaced by *Saccharomyces cerevisiae* [11]. Indeed, the role of non-*Saccharomyces* yeasts in the winemaking process has been investigated in recent years. Non-*Saccharomyces* yeasts can produce some extracellular enzymes to decompose the aroma precursor substances in wine to release aroma substances, and they can also metabolize glycerol, esters, and other products, which is beneficial to improve the flavor and sensory quality of the wine [12,13]. However, because of the low fermentation kinetic behavior and high sensitivity to ethanol of non-*Saccharomyces* yeasts, it is hard to ferment completely in the conditions of a pure starter culture [14]. Therefore, in combination with S. *cerevisiae* (the strain with strong fermentation ability and remarkable tolerance to high alcohol levels) [15], non-*Saccharomyces* yeast could significantly improve kiwi wine performance. For instance, *T. delbrueckii* can increase the glycerol content [16] and decrease the acetic acid of wine [17]; *K. thermotolerans* is known to produce high L-lactic acid and low volatile acidity [18]; the genus *Pichia* can reduce off-flavor production, such as acetaldehyde, and increase the content of polysaccharides which are positively associated with the smoothness of mouth-feel of the wine [19]. In addition, it was reported that yeast species have a huge impact on flavonoid and anthocyanins that affect wine color [20]. Nevertheless, there are relatively few studies on the influence of co-fermentation to a wine color. Furthermore, *S. cerevisiae* has been proven to reduce the phenolic content of wine via the adsorption of phenolic composition onto yeast cell walls, causing a negative influence on wine quality [21]. To the best of our knowledge, very limited research is available about the effect of co-fermentation on quality characteristics of kiwi wine.

Based on the above statement, in this study, different inoculations of *S. cerevisiae* with three non-*Saccharomyces* yeasts (*Torulaspora delbrueckii*, *Kluyveromyces thermotolerans*, *Pichia fermentans*) were designed to investigate the effects of co-fermentation on kiwi wine quality. Additionally, physicochemical characteristics, color parameters, phenolic profiles, antioxidant activities, organic acids, as well as taste sense of different kiwi wines were evaluated. This work comprehensively analyzed the influence of different non-*Saccharomyces* yeasts co-fermentation on the quality characteristics of kiwi wine, which will provide theoretical guidance for co-fermentation of non-*Saccharomyces*/*S. cerevisiae* and accelerate the industrialization process of kiwi wine.

## 2. Materials and Methods

### 2.1. Experimental Materials and Reagents

“Xuxiang” kiwifruit (soluble solid content, 13.7 °Brix; pH value, 3.18) were purchased from the orchard of Yangling (Shaanxi, China). *Torulaspora delbrueckii* A25, *Kluyveromyces thermotolerans* R69, and *Pichia fermentans* Z9Y were preserved in the College of Enology of Northwest A&F University. *Saccharomyces cerevisiae* WLP775 was preserved in the College of Food Science and Engineering of Northwest A&F University. Chemicals and reagents (≥98.0%, Solarbio Chemical Company, Beijing, China) used were high-performance liquid chromatography (HPLC) grade including gallic acid, protocatechuic acid, (+)-catechin, chlorogenic acid, (−)-epigallocatechin gallate (EGCG), (−)-epicatechin, (−)-gallocatechin gallate (GCG), caffeic acid, syringic acid, (−)-epicatechin gallate (ECG), *p*-coumaric acid, ferulic acid, hyperoside, phloridzin, and ellagic acid.

### 2.2. Winemaking and Experimental Design

Raw material (approximately 30 kg) with good maturity were squeezed with a laboratory juicer (HausElec, Qingdao, China) to obtain kiwi juice, and then the kiwi juice mixed with pomace were transferred to 10 glass fermentation cylinders with the loading volume of 2 L, SO_2_ (60 mg/L) and pectinase (50 mg/L) were added to each of glass fermentation cylinders. After 24 h of maceration at room temperature, yeasts were inoculated to start alcohol fermentation at 22 °C. The types, amounts, and sequences of inoculated yeasts under different treatments are shown in Table 1. Pure fermentation means that the fermentation was started by adding one kind of yeast at 2 × 10^6^ CFU/mL. Simultaneous co-fermentation means adding the *S. cerevisiae* and non-*Saccharomyces* yeasts simultaneously at 1 × 10^6^ CFU/mL, respectively. Sequential co-fermentation means adding non-*Saccharomyces* yeasts at 1 × 10^6^ CFU/mL to ferment first, then adding the *S. cerevisiae* at 1 × 10^6^ CFU/mL after 48 h. At the end of fermentation (reducing sugar concentration kept constant for three consecutive days), all the samples were centrifuged at 4 °C, 5000× *g* for 15 min, and the supernatant was taken and stored at −20 °C before analysis.

### 2.3. Yeast Growth Analysis

Yeast growth under different inoculation conditions was explored in model synthetic medium (MSM). The ingredients of MSM referred to Contreras et al. [22]. The group settings were the same as in Table 1. The number of yeast in different growth stages was measured by plate counting method to evaluate the growth of yeast in each treatment group in MSM.

### 2.4. Determination of Physicochemical and Color Parameters

Alcohol, reducing sugars and total acidity (TA) were determined according to methods described by Chinese Std GB/T 15038-2006 (Analytical methods of wine and fruit wine). Soluble solid contents (SSC) were tested in an Atago refractometer at 20 °C with values being expressed as °Brix, and the pH was measured using a pH meter, GLP 21 (Crison Instruments SA, Barcelona, Spain). The color parameters of kiwi wine were assayed according to methods proposed by Liu et al. [23]. Samples were scanned in a visible spectrum (400–700 nm) using a UV spectrophotometer (UV-2550, Shimadzu, Kyoto, Japan).

### 2.5. Determination of Total Phenolic Content

Total phenolic content (TPC) was determined according to the Folin–Ciocalteu method [24]. Firstly, 20-time diluted wine samples (1 mL) were added to the colorimetric tube. Then, 5 mL distilled water, 1 mL Folin–Ciocalteu, and 3 mL 7.5% sodium carbonate solution were added to the same colorimetrical tube in order. The mixture was kept at 22 °C for 2 h, and then the absorbance of samples was measured at 765 nm. TPC was calculated by its absorbance and corresponding standard curve (Y = 0.0211X + 0.0057, R^2^ = 0.9997), and the results were given as gallic acid equivalent (GAE) of kiwi wine. 

### 2.6. Analysis of Phenolic Composition

Phenolic compositions were tested by HPLC (LC-2030, Shimadzu, Kyoto, Japan) in accordance with the literature of Zhang et al. [25]. The phenolic compounds were extracted from the wine samples (25 mL) by ethyl acetate (3 × 20 mL) at pH 7.0 and pH 2.0, respectively. The ethyl acetate extracts were mixed and rotationally evaporated at 40 °C. Finally, the concentrated liquid was dissolved in 2.5 mL methanol. Then, all samples were filtered with membrane of an ester mixture 0.22 μm, and 20 μL was injected into the chromatograph. 

HPLC conditions were as follows: chromatographic column was ZORBAX SB-C_18_ column (4.6 × 250 mm, 5 μm, Agilent, Palo Alto, CA, USA) and the column temperature was maintained at 30 °C. The spectra were recorded from 190 to 800 nm, and the chromatograms were monitored at 280 and 320 nm. The gradient elution with eluent A (2% acetic acid in deionized water) and eluent B (methanol) was executed at the flow rate of 0.8 mL/min. The elution program was set to 0–10 min (5–30% B), 10–35 min (30–50% B), 35–40 min (50–60% B), 40–45 min (60–70% B), 45–50 min (70–5% B), and 50–55 min (5–5% B). Referring to the standard curve for each mono-phenol, the retention time and peak area were used for qualitative analysis and quantification analysis, respectively.

### 2.7. Analysis of Antioxidant Activities

The 1,1-diphenyl-2-picrylhydrazyl (DPPH) radical, hydroxyl radical (OH·), superoxide anion radical (O_2_•^−^) scavenging abilities were measured for a comprehensive evaluation of the antioxidant activities. The effect of the samples on DPPH radical was estimated according to Zengin et al. [26] with slight modification of the dilution for the wine sample. The scavenging activity for OH· was detected using the salicylate spectrophotometric method [7]. The measurement of O_2_•^−^ scavenging activity was carried out according to Lan et al. [27]. The samples were 20-fold diluted, 10-fold diluted, and not diluted in these three assays, respectively. All the data were reported as the rate of scavenging (%) at the same concentration and volume. 

### 2.8. Organic Acids Analysis

The organic acids in kiwi wine were qualitatively and quantitatively determined by HPLC, referring to the protocols by Endelmann et al. [28]. The wine sample was filtered twice with 0.22 μm filter membrane for the determination. The chromatographic column was ZORBAXSB-C_18_ (4.6 × 250 mm, 5 μm particle size, Agilent, Palo Alto, CA, USA). Chromatographic conditions were as follows: Column thermostat temperature (25 °C), detection wavelength (210 nm), and mobile phase (diammonium hydrogen phosphate solution, 0.01 mol/L). Elution was conducted in isocratic mode with a flow rate of 0.7 mL/min. The injection volume of each sample was 10 μL in triplicate. According to the standard curve of different organic acids, the retention time and peak area were used for qualitative and quantitative analysis, respectively.

### 2.9. Sensory Analysis

The sensory analysis of wine samples was carried out by quantitative descriptive analysis [29]. The panel was composed of 20 trained winemaking professionals (10 male and 10 female) between the ages of 24 and 35. All participants gave their permission to participate in this research. Wine samples were randomly coded and randomly assigned. The test was carried out at 25 °C, with a pour volume of 10 mL for each wine sample. The description of five taste attributes of kiwi wine aroma was defined as sour, bitter, sweet, astringent, and alcoholic. Each panelist rated the intensity of flavor features (0, no flavor features; 1, very weak; 2, weak; 3, medium; 4, strong; 5, very strong). The analysis was repeated three times for each wine sample.

### 2.10. Statistical Analysis

Data were expressed as means ± standard deviation in triplicate. Duncan’s test, at a significance level of *p* < 0.05, and one-way analysis of variance (ANOVA) were performed to compare the differences among samples. GraphPad Prism version 6.0 for Windows (GraphPad Software, San Diego, CA, USA) was used for plotting column graphs. Principal component analysis (PCA) and correlation analysis were performed using SPSS 20.0 (Chicago, IL, USA) and GraphPad Prism version 6.0, respectively. The visualization of the results was performed using TBtools (v2.056) software.

## 3. Results

### 3.1. Physicochemical Characteristics of Kiwi Wine

Physicochemical values of the wine samples are shown in Table 2. All wine samples ranged in alcohol content from 3.62% to 4.93%, with the highest being the WLP755 group at 4.93%. Soluble solid contents and reducing sugars were around 6.00–6.42%, 5.57–5.96 g/L, respectively. The total acid content of the WLP755 group was 15.75 g/L. Total acid contents of the R69-related groups (R69, R69-D0 and R69-D2) and the Z9Y-related groups (Z9Y, Z9Y-D0 and Z9Y-D2) were lower than that of the WLP775 group. However, total acid contents of the A25-related group (A25, A25-D0 and A25-D2) were higher than that of the WLP775 group. No significant difference was observed among the treatments regarding pH value.

### 3.2. Dynamic Change in Yeast Growth

**Figure 1:**
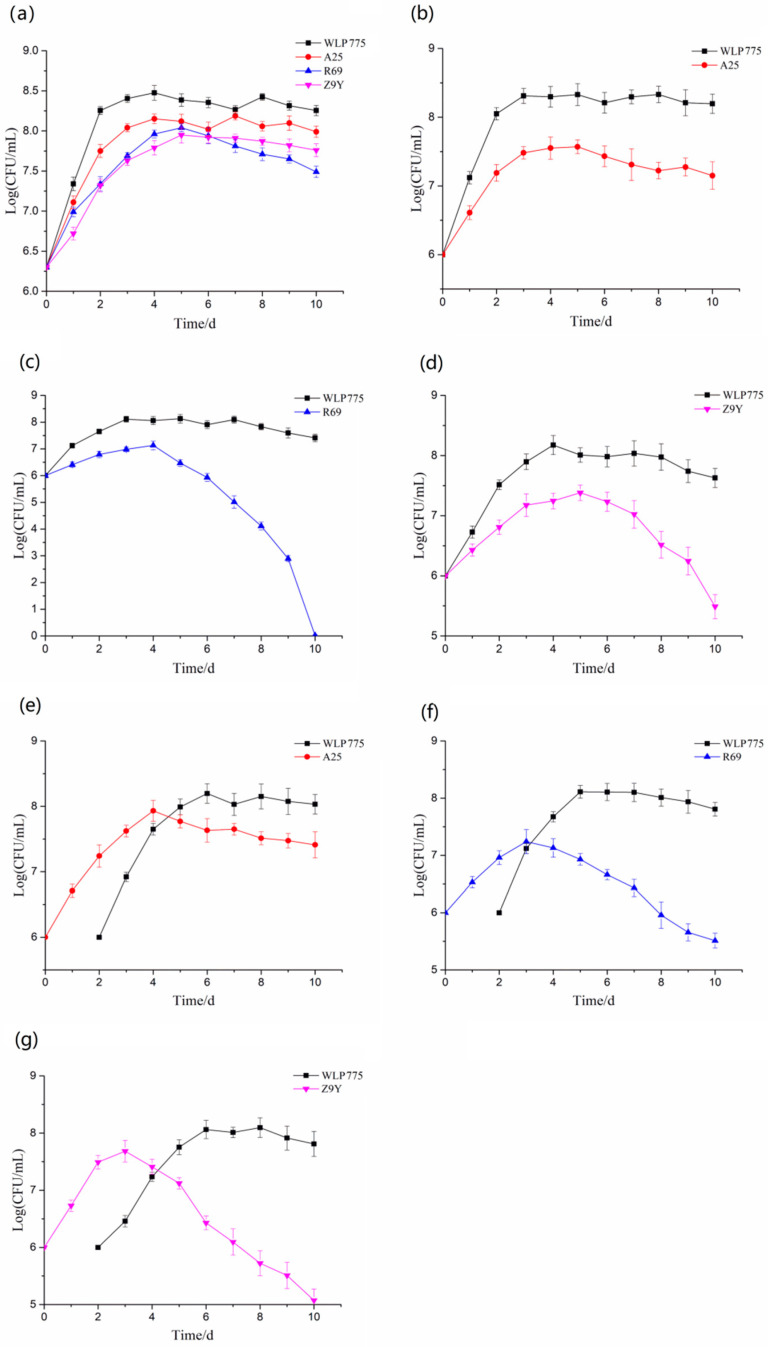
Dynamic curves of the count of yeasts in MSM. (**a**) showed the dynamic curves of the count of four yeast strains during single fermentation, and the inoculated amount was 2 × 10^6^ CFU/mL. (**b**–**d**) showed the dynamic curves of the count of *T. delbrueckii* A25, *K. thermotolerans* R69 and *P. fermentans* Z9Y simultaneously inoculated with *S. cerevisiae* WLP775, respectively, and the inoculated amount was 1 × 10^6^ CFU/mL for each strain. (**e**–**g**) showed the dynamic curves of the count of *T. delbrueckii* A25, *K. thermotolerans* R69, and *P. fermentans* Z9Y sequentially inoculated with *S. cerevisiae* WLP775, respectively, and the inoculated amount was 1 × 10^6^ CFU/mL for each strain.

The results of single-strain fermentation (Figure 1a) showed that *S. cerevisiae* WLP775 entered a stable phase in MSM on day 2, and the yeast concentration reached the maximum value (2.98 × 10^8^ CFU/mL) on day 4 of fermentation., while *K. thermotolerans* R69, *T. delbrueckii* A25, and *P. fermentans* Z9Y entered the stable phase at the 3rd, 4th, and 5th days of fermentation, respectively. Their maximum bacterial counts were between 8.91 × 10^7^ CFU/mL and 1.42 × 10^8^ CFU/mL. When the *S. cerevisiae* and non-*Saccharomyces* yeasts were inoculated at the same time, the growth of the three types of non-*Saccharomyces* yeasts would be inhibited to different degrees. *K. thermotolerans* R69 was the most inhibited, with the highest bacterial count of 1.35 × 10^7^ CFU/mL. As can be seen from Figure 1e–g, when sequentially inoculated, *S. cerevisiae* WLP755 inhibited the growth of three non-*Saccharomyces* yeasts more significantly.

### 3.3. Color Parameters of Kiwi Wine

The mean values of the CIE L*a*b* parameters among these kiwi wines are shown in Table 3. Compared with WLP775, the lightness (L*) value of the other samples was all increased, and the highest L* value was detected in Z9Y-D2, which was 99.92. All samples had a negative red-greenness (a*) value and positive yellow-blueness (b*) value, indicating that all wines were yellow-green. From the perspective of inoculation method, a higher a* value and higher b* value were tested in Z9Y-D0 (a*, −1.03; b*, 5.00) compared with Z9Y-D2 (a*, −0.92; b*, 4.28), which suggested that the simultaneous co-fermentation of kiwi wine with *P. fermentans/S. cerevisiae* had a higher yellow tone and higher green tone. The R69-D0 group and R69-D2 group had higher Chroma (c*) values, with values of 6.19 and 6.08, respectively. In addition, the ΔEab* values of all the treatment groups were greater than 1.0 and were distributed in the range of 1.47–2.53.

### 3.4. TPC and Antioxidant Activities of Kiwi Wine

**Figure 2:**
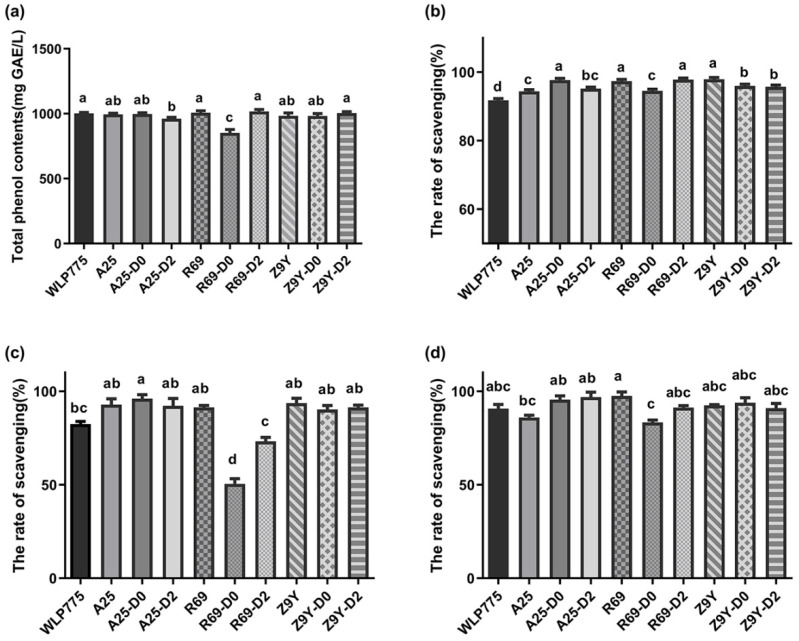
The total phenolic contents and antioxidant activities of 10 treatments. (**a**) Total phenolic contents (TPC); (**b**) DPPH radical scavenging ability; (**c**) hydroxyl radical scavenging ability; (**d**) superoxide anion radical scavenging ability. Different lowercase letters on the bar charts indicate. Significant Differences (*p* < 0.05).

As shown in Figure 2a, the TPC of the kiwi wine samples ranged from 851.30 to 1016.80 mg GAE/L, and the TPC of the R69-D0 group was the lowest and found to be significantly lower than that of the other groups. In addition, it can be seen from Figure 2b–d that there are significant differences in the antioxidant activities among all the treatments. The scavenging effect of DPPH on the kiwi wine samples was similar to the hydroxyl radical scavenging ability. The maximum scavenging ability rates were 98.04% and 97.50%, respectively, both of which appeared in the A25-D0 group. The superoxide radical scavenging ability was the highest in the R69 group (98.95%) and the lowest in the R69-D0 group (85.35%).

### 3.5. Phenolic Profiles of Kiwi Wine

**Figure 3:**
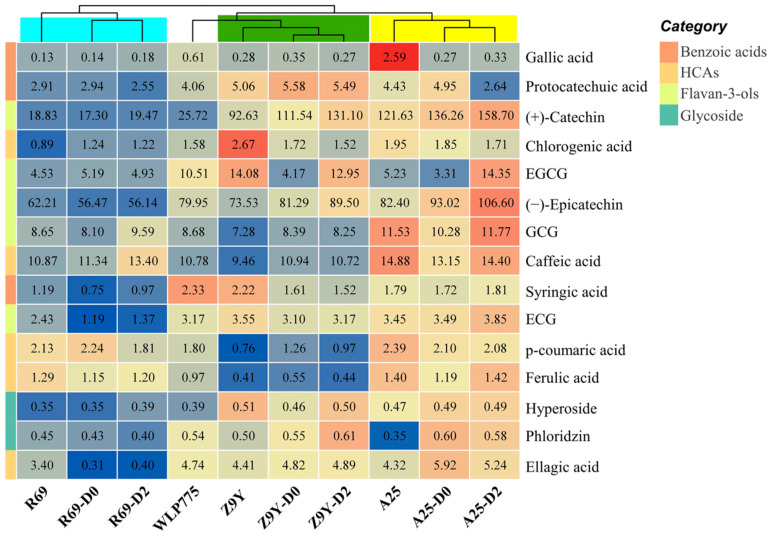
Group clustering and heat map visualization of phenolic compounds for kiwi wines with different treatments. The numbers in the color block represent the content. The difference of the color block in the same row represents the concentration difference of the compound in different groups.

Fifteen phenolic individual compounds were identified (Figure 3 and Supplementary Data Table S1). The total contents of the tested phenolics (Σ phenols) of wines among A25- and Z9Y-related experimental groups were significantly higher than that of the WLP775 group (155.82 mg/L). Three kinds of benzoic acids were detected (gallic acid, protocatechuic acid, and syringic acid). The maximum values of these three benzoic acids were in group A25 (2.59 mg/L), group Z9Y-D0 (5.58 mg/L) and group WLP775 (2.33 mg/L), respectively. The benzoic acid concentrations of samples treated by three non-*Saccharomyces* strains were remarkably different from WLP775. Among the five kinds of hydroxycinnamic acids (HCAs), the highest contents of four HCAs were found in the A25-related groups. The caffeic acid content is higher than the other four HCAs, especially in the A25-D2 group, the caffeic acid content reached 14.40 mg/L. In this study, flavan-3-ols (catechins, epicatechins, EGCG, GCG, ECG) accounted for 79.08–91.29% of the polyphenols in all treatments. Catechin was determined to be the most abundant flavan-3-ols in the A25- and Z9Y-related groups. In addition, there was no significant difference in regard to the concentration of hyperoside and phloridzin among the different treatments.

### 3.6. Principal Component Analysis

**Figure 4:**
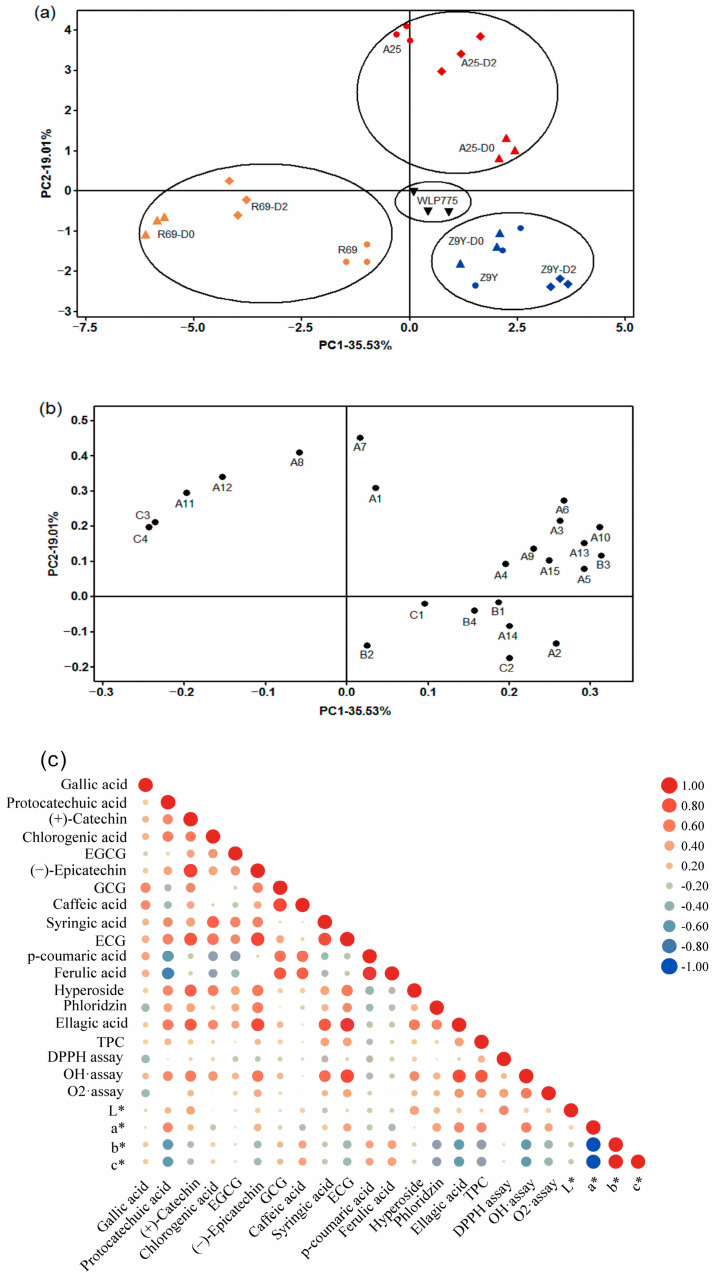
PCA and correlation analysis of different fermentation treatments as a function of phenolics contents, antioxidant activity and color parameter of kiwi wines. (**a**) classification of all treatments analyzed in functions of PC1 and PC2; (**b**) PCA loading value of all analyzed parameters. Abbreviations: A1 = gallic acid, A2 = protocatechuic acid, A3 = (+)-catechin, A4 = chlorogenic acid, A5 = EGCG, A6 = (−)-epicatechin, A7 = GCG, A8 = caffeic acid, A9 = syringic acid, A10 = ECG, A11 = *p*-coumaric acid, A12 = ferulic acid, A13 = hyperoside, A14 = phloridzin, A15 = ellagic acid, B1 = TPC, B2 = DPPH assay, B3 = OH· assay, B4 = O_2_•^−^ assay, C1 = L*, C2 = a*, C3 = b*, C4 = c*. (**c**) the correlation of color, phenolic compositions, TPC, and antioxidant activities.

For further distinguishing of the different treatment wines, PCA was performed to discriminate several special parameters (phenolic compositions, antioxidant activities, and chromatic characteristics) of the samples. Figure 4a,b showed the scoring and loading diagrams. In this study, the first two principal components (PCs) accounted for 54.54% of variance (35.53% for PC1 and 19.01% for PC2). All the samples were mainly located in three quadrants. There was a good distinction between different treatment groups. As shown in Figure 4a, the 10 treatments were grouped into four clusters based on selected yeast strains. The WLP775 wine sample was located around the origin and distinguished from other wines. A25-related groups were located at the positive end of PC2 and showed a higher value of specific phenolic compounds, b* and c*. Z9Y-related groups were located at the positive end of PC1, characterized by L*, a*, higher TPC, and stronger antioxidant activities, while R69-related groups showed weak relationship with most tested parameters. The TPC (B1), antioxidant activities (B2, B3, B4), and the phenolics except for caffeic acid (A8), *p*-coumaric acid (A11), and ferulic acid (A12) were positively associated with PC1 from the factor loading plot (Figure 4b), and the A25-D0, A25-D2, Z9Y, Z9Y-D0 and Z9Y-D2 wine samples at the positive end of PC1 (Figure 4b) were correlated with these variables.

### 3.7. Correlation Analysis

For the sake of exploring the influence of phenolic compounds on antioxidant ability and color features in kiwi wine, the correlation among antioxidant activities, chromatic characteristics, and individual phenolic composition were measured. Pearson’s linear coefficients were calculated, and the results are shown in Figure 4c. Significant correlation was found between the OH· scavenging ability and O_2_•^−^ scavenging value (r = 0.549, *p* < 0.01), and both OH· and O_2_•^−^ scavenging values were significantly correlated to TPC (r = 0.761, r = 0.449; *p* < 0.01, *p* < 0.05, respectively). Moreover, OH· scavenging ability was significantly relevant to protocatechuic acid, (+)-catechin, (−)-epicatechin, syringic acid, ECG, hyperoside, and ellagic acid contents (*p* < 0.01), respectively. Meanwhile, the positive correlation of ECG and ellagic acid with O_2_•^−^ scavenging ability was presented (r = 0.368, r = 0.442; *p* < 0.05, respectively). In addition, TPC was significantly correlated to syringic acid, ECG, and ellagic acid (r = 0.392, r = 0.406, r = 0.419; *p* < 0.05, respectively). With regard to the effect of phenolic composition on kiwi wine color, lightness (L*) showed the strong positive relationship with (+)-catechin (r = 0.381, *p* < 0.05) and hyperoside (r = 0.424, *p* < 0.05). On the other hand, the TPC and OH· scavenging ability presented significantly positive correlation with a* (r = 0.509, r = 0.540; *p* < 0.01, respectively), indicating that green color intensity (a*) reflected the abundance of antioxidants and phenolic compounds in kiwi wine. 

### 3.8. Organic Acids Analysis

In this study, seven organic acids were detected in ten different kiwi wines, including oxalic acid, tartaric acid, malic acid, lactic acid, acetic acid, citric acid, and succinic acid. According to Table 4, the main organic acids in kiwi wine were citric acid, tartaric acid, and malic acid, and the concentration ranges were 8183.7–10,046.54 mg/L, 2461.74–3074.18 mg/L, 1832.03–2350.29 mg/L, respectively. The organic acids with lower content were oxalic acid and acetic acid, with concentrations ranging from 120.11 to 139.53 mg/L and 37.33 to 73.36 mg/L. Except lactic acid and succinic acid, the highest concentrations of the other five organic acids were found in A25-related groups. The highest concentrations of lactic acid and succinic acid were detected in Z9Y-related groups. It is worth mentioning that the highest and lowest concentrations of lactic acid both appeared in the Z9Y-related groups, with values of 454.37 mg/L (group Z9Y) and 310.27 mg/L (group Z9Y-D0), respectively.

### 3.9. Sensory Analysis

**Figure 5:**
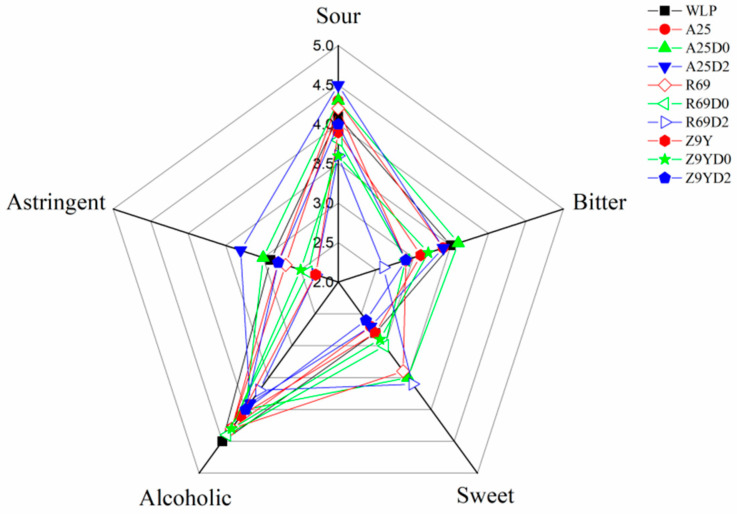
Sensory analysis of kiwi wines with different treatments.

The sensory analysis results of the kiwi wine samples are shown in Figure 5. In all the treatments, the main tastes were alcoholic and sour, followed by sweet and astringent. Specifically, group WLP775 scored highest for the alcohol taste. The acidity of A25-D0 and A25-D2 was slightly greater than the taste of alcohol. In addition, the A25-D0 group had the highest bitterness score, followed by the WLP775 group. In terms of sweetness characteristics, the R69-D2, A25-D0, and R69 groups had higher sweetness scores than the other groups.

## 4. Discussion

It has been reported that the inoculation of some non-*Saccharomyces* yeasts in co-fermentations with *S. cerevisiae* was able to reduce the alcohol concentration in wine [22]. Similar results were found in this study as the alcohol content of the co-fermented kiwi wine sample was significantly lower than that of the WLP775 group. The reducing sugars of the wines fermented by simultaneous co-fermentation was dramatically lower than that of sequential co-fermentation, indicating that *S. cerevisiae* had stronger fermentation capacities than the non-*Saccharomyces* yeasts to withstand the harsh environmental conditions in winemaking. Therefore, simultaneous inoculation resulted in the rapid consumption of nutrients in the early stage of fermentation. For total acid content, the results were consistent with the previous data that some non-*Saccharomyces* yeast in co-fermentations with *S. cerevisiae* reduced the acid concentration in the wine [30], while some could produce more acid like succinic acid [16], which was attributed to the differences in metabolism in three non-*Saccharomyces* yeasts. Thus, the results indicated that the effects of *K. thermotolerans* R69 and *P. fermentans* Z9Y on the content of alcohol and total acidity were different from *T. delbrueckii* A25 of kiwi wine.

Monitoring the variation of the number of *S. cerevisiae* and non-*Saccharomyces* yeasts is helpful to understand their interaction in co-fermentation. On the whole, *S. cerevisiae* had better adaptability and rapid growth in MSM. In the three groups of simultaneous co-fermentation, the growth of non-*Saccharomyces* yeasts was inhibited in the middle and late stages of fermentation, which may be due to the competitive inhibition of *S. cerevisiae* and non-*Saccharomyces* yeasts for glucose, amino acids, and other nutrients in the same fermentation system. *S. cerevisiae* showed a better growth trend due to its greater tolerance of metabolites, such as alcohol. In the three groups of sequential co-fermentation, the growth of non-*Saccharomyces* yeasts rapidly entered the logarithmic phase in the first two days due to sufficient nutrients. After inoculation with *S. cerevisiae*, the growth of non-*Saccharomyces* was still on the rise because the number of non-*Saccharomyces* yeasts was dominant. However, on the 4th and 5th day of fermentation, due to the stronger tolerance of *S. cerevisiae* to the stressed environment, its growth gradually dominated, while significantly inhibiting the growth of non-*Saccharomyces* yeasts. Studies have shown that in a co-fermentation system, *S. cerevisiae* can produce toxins to form homicidal inhibition on other yeasts in the environment [31]. In conclusion, there are differences in the interaction between *S. cerevisiae* and non-*Saccharomyces* yeasts with different inoculation time and order, which may be the potential reason for affecting the quality of fruit wine produced by mixed fermentation.

In general, non-*Saccharomyces* yeasts have higher pectinase activity. For example, the *Torulaspora* genus has been recorded to generate abundant enzymes like pectinases, polygalacturonase, amylases, and proteases, which are beneficial to the clarification and filtration process during winemaking [32]. This is also the main reason for the increase in lightness of kiwi wine fermented by non-*Saccharomyces* yeasts. Simultaneous inoculation of *P. fermentans* and *S. cerevisiae* facilitates *P. fermentans* to produce more metabolites affecting color. Previous studies reported that co-fermentation of *P. fermentans*/*S. cerevisiae* could enhance the synthesis of polymeric anthocyanins, which were important for color stability [33]. Chroma (c*) value is a parameter to indicate the contribution of a* (redness) and b* (yellowness). The chroma (c*) value of kiwi wine in the R69-D0 and R69-D2 groups was significantly increased in comparison with the WLP775 group, suggesting that more color vividness appeared in kiwi wines co-fermented with *K. thermotolerans*/*S. cerevisiae*. There were significant differences in hue (H*) between the pure fermentation samples and sequential co-fermentation samples (A25 and A25-D2, Z9Y and Z9Y-D2, respectively), indicating that the inoculation method changed the hue value of kiwi wine. In addition, chromatism between the WLP775 group and other samples can be confirmed by calculating the color difference values (ΔE_ab_*). Normally, human eyes can discriminate between two colors when ΔE_ab_* ≥ 1 [34]. Therefore, in this study, non-*Saccharomyces* yeasts caused significant color differences in kiwi wine.

All kiwi wine samples showed good antioxidant capacity. The DPPH radical scavenging rates of wines treated with non-*Saccharomyces* yeasts were significantly higher than those of WLP775 kiwi wine. Hence, it is reasonable to infer that co-fermentation with non-*Saccharomyces* yeast improved the DPPH scavenging ability of kiwi wine. Antioxidant activity is attributed to the hydroxyl groups of phenolics. Specifically, the effect of antioxidants on DPPH radicals resulted from their hydrogen-donating ability [35]. The highest scavenging percentage of O_2_•^−^ was detected in R69 wine; however, the lowest was found in R69-D0. This result may be attributed not only to the effects of non-*Saccharomyces*/*S. cerevisiae* on phenolics in kiwi wine but to the complex interaction products of yeasts, and the interactions differed considerably between *S. cerevisiae* and different non-*Saccharomyces* yeasts [36].

Phenolic compounds have great benefits for health. They can react with reactive oxygen species, such as superoxide anion radicals and hydroxyl radicals, and inhibit lipid oxidation at an early stage [37]. In this study, co-fermentation increased the concentrations of the phenolic compounds in wine except the R69-related groups, which was consistent with previous research results [38]. In the relevant studies of red wine, gallic acid was considered to be the most important benzoic acid [39], which was different from the results in the kiwi wine in this study. Hydroxycinnamic acid is one of the most representative phenolic acid compounds in fruits and their wines and has good antioxidant potential [40]. The results in this study showed that the contents of HCAs in the A25-related groups were significantly higher than in the WLP775 group, indicating that the co-fermentation of *T. delbrueckii* A25 and *S. cerevisiae* WLP775 enhanced the antioxidant capacity of kiwi wine, which was also confirmed by the subsequent determination of antioxidant activity. Co-fermentation of kiwi wine by *S. cerevisiae* and non-*Saccharomyces* yeasts (*T. delbrueckii* and *P. fermentans*) dramatically increased the contents of flavan-3-ols in kiwi wine. Increased concentrations could be attributed to the breakdown of plant cell walls by yeasts, which releases phenolic compounds [41]. However, the contents of flavan-3-ols in R69-related groups (treated with *K. thermotolerans*) were not increased. The reason for these differences was due to the fermentation characteristics of different yeast strains.

The results of PCA indicated that wines fermented with different yeasts and inoculation methods were accurately separated, which was influenced mainly by antioxidant activities and a majority of the tested phenolic compounds in the kiwi wines. Meanwhile, simultaneous co-fermentation samples were separated from sequential co-fermentation across PC1. Roughly, PC2 separated wines into two parts, one part included the A25 group, which was influenced positively by gallic acid (A1), GCG (A7) and caffeic acid (A8), and the other was distributed at the negative end of PC2, which was affected by L* and a*. The results indicated that kiwi wine samples fermented by *K. thermotolerans* and *P. fermentans* had higher lightness and greenness value. A possible explanation for this result was that non-*Saccharomyces* yeasts produced metabolites that affect color during alcoholic fermentation. Previous studies reported that co-fermentation with non-*Saccharomyces* yeast can enhance the synthesis of certain metabolites (i.e., acetaldehyde, pyruvic acid and vinylphenols) that were important for color stability in red wines [42].

According to the results of the correlation analysis, antioxidant activities were positively related to TPC, confirming the previous findings [43]. Flavonoids were effective antioxidants due to the inhibition of O_2_•^−^ production. In addition, three phenolic compounds, syringic acid, ECG and ellagic acid, played a major role in the antioxidant capacity of the kiwi wine.

Organic acids can directly affect the sensory evaluation of the kiwi wine taste. Organic acids can be converted into aromatic esters during the aging process, and the appropriate amount of organic acids in fruit wine can make the wine taste mellow and harmonious and can inhibit the growth of harmful bacteria to a certain extent [44]. Citric acid is the organic acid with the highest content among all kiwi wine samples. Citric acid has a refreshing, fresh sour taste, but the aftertaste lasts for a short time. Citric acid not only comes from fresh kiwifruit but can also be produced during the fermentation process. Tartaric acid has an astringent and strong sour taste. In this study, there is no significant difference in the content of tartaric acid in the kiwi wine among all treatments. During the fermentation process, the tartaric acid in wine will not be metabolized, but it can form tartrate precipitation with potassium and calcium ions, which can make the wine more stable and clarified. Acetic acid is a product of yeast metabolism, mainly produced in the early stages of fermentation, and is the main component of volatile acids. However, high concentrations of acetic acid can affect the balance of the wine and cause a bitter taste. In this study, the acetic acid contents of the kiwi wine in all the treatment groups were very low, and the difference among the treatments was not significant. On the whole, the kiwi wines of the A25-D0 and A25-D2 groups were rich in flavor substances, including the sour, sweet, and astringent taste in addition to the alcoholic taste, which enhanced the mellow feeling of the kiwi wine.

## 5. Conclusions

In conclusion, the results of this study indicated that different co-fermentation methods of three non-*Saccharomyces* yeasts and *S. cerevisiae* differently affected the alcohol production, TA, color, phenolic profile, and organic acids of the kiwi wines. Application of *T. delbrueckii* was superior to *K. thermotolerans* or *P. fermentans* in improving the quality of the “Xuxiang” kiwi wine. Meanwhile, compared to simultaneous inoculation, sequential inoculation was a better co-fermentation method as it increased the antioxidant abilities and phenolic profiles of the kiwi wine. PCA indicated that wines fermented with different yeasts were distinguished by phenolic composition, color, and antioxidant activities. Furthermore, the significant correlations among TPC and antioxidant, individual phenolic compounds or color intensity was confirmed. This study has a positive meaning to broaden research on the influence of non-*Saccharomyces* yeast and its co-fermentation with *S. cerevisiae* on the quality characteristics of kiwi wine.

## Figures and Tables

**Table 1:** The experimental design of kiwi wine fermentation.

Yeast	Pure Fermentation	Simultaneous Co-Fermentation	Sequential Co-Fermentation
*S. cerevisiae*	WLP775	-	-
*T. delbrueckii*	A25	A25-D0	A25-D2
*K. thermotolerans*	R69	R69-D0	R69-D2
*P. fermentans*	Z9Y	Z9Y-D0	Z9Y-D2

**Table 2:** Physicochemical characteristics of kiwi wines resulting from different inoculations.

Wine Samples	Alcohol (% *v*/*v*)	SSC (%)	Total Reducing Sugar (g/L)	pH	Total Acidity (g/L)
WLP775	4.93 ± 0.35 a	6.31 ± 0.30 ab	5.85 ± 0.11 abc	3.18 ± 0.01 a	15.75 ± 0.23 c
A25	4.38 ± 0.11 bc	6.42 ± 0.17 a	5.95 ± 0.13 a	3.19 ± 0.01 a	16.14 ± 0.08 b
A25-D0	3.62 ± 0.08 e	6.08 ± 0.08 b	5.66 ± 0.13 cde	3.20 ± 0.04 a	16.11 ± 0.11 b
A25-D2	3.72 ± 0.07 de	6.17 ± 0.08 ab	5.96 ± 0.08 a	3.18 ± 0.03 a	16.45 ± 0.10 a
R69	4.18 ± 0.22 c	6.11 ± 0.25 b	5.74 ± 0.07 bcde	3.18 ± 0.02 a	13.99 ± 0.07 e
R69-D0	4.33 ± 0.07 bc	6.13 ± 0.09 b	5.64 ± 0.12 de	3.21 ± 0.04 a	14.27 ± 0.13 d
R69-D2	3.90 ± 0.07 d	6.00 ± 0.13 b	5.89 ± 0.17 ab	3.20 ± 0.05 a	14.21 ± 0.21 d
Z9Y	3.79 ± 0.11 de	6.09 ± 0.08 b	5.80 ± 0.05 abcd	3.17 ± 0.03 a	13.10 ± 0.09 g
Z9Y-D0	4.52 ± 0.09 b	6.04 ± 0.11 b	5.57 ± 0.10 e	3.21 ± 0.04 a	13.85 ± 0.08 e
Z9Y-D2	3.81 ± 0.08 de	6.13 ± 0.05 b	5.88 ± 0.04 ab	3.19 ± 0.03 a	13.46 ± 0.08 f

Note: Data showed the average of triplicates ± SD. Different letters within columns indicated differences among wine samples determined by the Duncan test at 95% confidence level. Total reducing sugar was expressed as g/L of glucose. TA was expressed as g/L of tartaric acid (g TA/L).

**Table 3:** The CIE L*a*b* parameters of different kiwi wines.

Wine Samples	L*	a*	b*	c*	H*	ΔE_ab_*
WLP775	97.55 ± 0.57 b	−1.05 ± 0.05 d	5.16 ± 0.22 c	5.26 ± 0.22 c	−1.37 ± 0.00 bc	-
A25	99.00 ± 0.79 a	−1.11 ± 0.03 c	5.68 ± 0.15 d	5.78 ± 0.15 b	−1.38 ± 0.01 d	1.54 *
A25-D0	99.57 ± 0.26 a	−1.03 ± 0.01 de	4.95 ± 0.02 bc	5.06 ± 0.02 cd	−1.37 ± 0.01 b	2.03 *
A25-D2	98.96 ± 0.41 a	−1.16 ± 0.01 bc	5.69 ± 0.02 d	5.81 ± 0.02 b	−1.37 ± 0.02 bc	1.51 *
R69	99.09 ± 0.69 a	−0.98 ± 0.01 f	4.75 ± 0.08 b	4.85 ± 0.08 d	−1.37 ± 0.01 bc	1.60 *
R69-D0	98.69 ± 0.76 ab	−1.22 ± 0.04 a	6.07 ± 0.22 e	6.19 ± 0.22 a	−1.37 ± 0.02 bc	1.47 *
R69-D2	99.08 ± 0.91 a	−1.19 ± 0.05 ab	5.96 ± 0.22 e	6.08 ± 0.22 a	−1.37 ± 0.00 c	1.73 *
Z9Y	98.97 ± 1.06 a	−1.17 ± 0.05 ab	5.63 ± 0.2 d	5.75 ± 0.20 b	−1.37 ± 0.00 b	1.50 *
Z9Y-D0	99.14 ± 0.79 a	−1.03 ± 0.02 de	5.00 ± 0.05 bc	5.10 ± 0.05 cd	−1.37 ± 0.01 bc	1.60 *
Z9Y-D2	99.92 ± 0.41 a	−0.92 ± 0.02 f	4.28 ± 0.02 a	4.38 ± 0.02 e	−1.36 ± 0.01 a	2.53 *

Note: All data were displayed as mean ± SD (*n* = 3). Data in the same column with different letters were statistically significant differences (*p* < 0.05) according to Duncan’s test. Abbreviations: L*, lightness; a*, red-greenness; b*, yellow-blueness; c*, chroma; H*, hue; ΔE_ab_*, total color differences between treatments and control.

**Table 4:** The organic acids in kiwifruit wines.

Wine Samples	Oxalic Acid (mg/L)	Tartaric Acid (mg/L)	Malic Acid (mg/L)	Lactic Acid (mg/L)	Acetic Acid (mg/L)	Citric Acid (mg/L)	Succinic Acid (mg/L)
WLP775	123.22 ± 7.13 bc	2731.86 ± 112.46 ab	2098.18 ± 46.48 ab	386.99 ± 21.72 c	59.26 ± 13.26 abc	9174.76 ± 352.87 abc	468.68 ± 20.94 bc
A25	131.62 ± 7.95 abc	2943.20 ± 105.80 ab	2345.40 ± 28.18 a	400.16 ± 6.02 bc	43.70 ± 7.96 c	9308.02 ± 457.67 abc	435.00 ± 17.86 bc
A25-D0	139.53 ± 6.93 a	3074.18 ± 163.61 a	2280.82 ± 47.58 a	408.87 ± 25.47 bc	73.36 ± 0.35 a	9689.68 ± 137.13 ab	487.24 ± 33.58 bc
A25-D2	125.46 ± 7.19 abc	3002.64 ± 148.49 ab	2350.29 ± 131.08 a	430.03 ± 25.05 ab	54.65 ± 2.75 abc	10,046.54 ± 61.15 a	407.17 ± 18.34 bc
R69	120.11 ± 7.52 c	2725.82 ± 62.03 ab	1922.07 ± 30.38 b	402.61 ± 16.53 bc	43.62 ± 21.01 c	9364.73 ± 218.29 abc	521.31 ± 27.58 bc
R69-D0	131.05 ± 6.89 abc	2605.46 ± 11.49 ab	1892.05 ± 68.65 b	343.54 ± 34.61 d	67.70 ± 19.67 ab	9195.26 ± 114.81 abc	365.48 ± 29.45 bc
R69-D2	121.99 ± 4.73 c	2461.74 ± 43.66 ab	1861.78 ± 33.57 b	341.86 ± 6.21 d	37.33 ± 5.76 c	9383.76 ± 280.98 abc	325.55 ± 9.08 c
Z9Y	138.97 ± 12.14 a	2998.39 ± 93.76 ab	2039.46 ± 25.44 ab	454.37 ± 21.41 a	42.56 ± 9.10 c	8714.98 ± 300.00 abc	566.70 ± 24.33 ab
Z9Y-D0	137.26 ± 5.44 ab	2842.60 ± 61.69 ab	1793.44 ± 32.63 b	310.27 ± 2.76 d	42.85 ± 8.28 c	8183.70 ± 211.99 c	746.39 ± 37.61 a
Z9Y-D2	133.83 ± 7.90 abc	2740.79 ± 50.40 ab	1832.03 ± 38.36 b	402.94 ± 21.78 bc	50.47 ± 7.27 bc	8312.55 ± 120.00 bc	738.98 ± 52.99 a

Note: Results were expressed as mean ± SD (*n* = 3). Data of same organic acids with different letters were statistically significant differences (*p* < 0.05) according to Duncan test.

## Data Availability

The original contributions presented in the study are included in the article/Supplementary Materials, further inquiries can be directed to the corresponding author.

## Supplementary Materials

The following supporting information can be downloaded at: https://www.mdpi.com/article/10.3390/foods13162599/s1, Table S1. The concentration of phenolic compounds in wine samples.
