# Supplementary material for: Effects of Different Non-Saccharomyces Strains in Simultaneous and Sequential Co-Fermentations with Saccharomyces cerevisiae on the Quality Characteristics of Kiwi Wine
Source: Foods. 2024 Aug 20;13(16):2599. doi: 10.3390/foods13162599 (PMC11353757; doi:10.3390/foods13162599)
Supplement: Supplementary file 1 [file foods-13-02599-s001.zip › foods-3141846-supplementary.pdf]

Table S1. The concentrations of phenolic compounds in wine samples.

phenolic compounds (mg/L)	WLP775	A25	A25-D0	A25-D2	R69	R69-D0	R69-D2	Z9Y	Z9Y-D0	Z9Y-D2
Gallic acid	0.61±0.05b	2.59±0.1a	0.27±0.02c	0.33±0.05c	0.13±0d	0.14±0.01d	0.18±0.03d	0.28±0.05c	0.35±0.07c	0.27±0.06c
Protocatechuic acid	4.06±0.17d	4.43±0.12c	4.95±0.07b	2.64±0.08f	2.91±0.14e	2.94±0.17e	2.55±0.11f	5.06±0.17b	5.58±0.08a	5.49±0.2a
(+)-Catechin	25.72±1.73f	121.63±3.68c	136.26±4.1b	158.70±5.89a	18.83±0.87g	17.30±0.83g	19.47±0.88g	92.63±2.25e	111.54±3.8d	131.10±3.32b
Chlorogenic acid	1.58±0.02de	1.95±0.05b	1.85±0.11bc	1.71±0.11cd	0.89±0.1g	1.24±0.08f	1.22±0.09f	2.67±0.04a	1.72±0.09cd	1.52±0.12e
EGCG	10.51±0.46c	5.23±0.11d	3.31±0.08g	14.35±0.62a	4.53±0.18ef	5.19±0.2d	4.93±0.14de	14.08±0.27a	4.17±0.06f	12.95±0.7b
(−)-Epicatechin	79.95±1.67c	82.40±3.15c	93.02±3.68b	106.60±3.31a	62.21±3.8e	56.47±0.88f	56.14±2.07f	73.53±4.08d	81.29±2.25c	89.50±4.1b
GCG	8.68±0.21d	11.53±0.31a	10.28±0.19b	11.77±0.32a	8.65±0.72d	8.10±0.16d	9.59±0.29c	7.28±0.21e	8.39±0.12d	8.25±0.12d
Caffeic acid	10.78±0.29cd	14.88±0.65a	13.15±0.85b	14.40±1.27ab	10.87±0.77cd	11.34±1.1c	13.40±1.01ab	9.46±0.88d	10.94±0.74cd	10.72±0.64cd
Syringic acid	2.33±0.14a	1.79±0.07b	1.72±0.08b	1.81±0.09b	1.19±0.09c	0.75±0.07d	0.97±0.12cd	2.22±0.08a	1.61±0.07b	1.52±0.52b
ECG	3.17±0.08c	3.45±0.1b	3.49±0.12b	3.85±0.07a	2.43±0.07d	1.19±0.05f	1.37±0.06e	3.55±0.1b	3.10±0.09c	3.17±0.12c
p-coumaric acid	1.80±0.08c	2.39±0.08a	2.10±0.1b	2.08±0.09b	2.13±0.1b	2.24±0.13ab	1.81±0.09c	0.76±0.06f	1.26±0.07d	0.97±0.07e
Ferulic acid	0.97±0.11c	1.40±0.1a	1.19±0.09b	1.42±0.11a	1.29±0.1ab	1.15±0.02b	1.20±0.1b	0.41±0.08d	0.55±0.04d	0.44±0.05d
Hyperoside	0.39±0.05bcd	0.47±0.07ab	0.49±0.06ab	0.49±0.06ab	0.35±0.03d	0.35±0.04cd	0.39±0.08bcd	0.51±0.08a	0.46±0.04abc	0.50±0.05a
Phloridzin	0.54±0.06abc	0.35±0.04d	0.60±0.19ab	0.58±0.06ab	0.45±0.06abcd	0.43±0.05bcd	0.40±0.07cd	0.50±0.1abcd	0.55±0.08abc	0.61±0.08a
Ellagic acid	4.74±0.62b	4.32±0.07bc	5.92±0.13a	5.24±0.6ab	3.40±0.53c	0.31±0.03d	0.40±0.04d	4.41±0.79bc	4.82±1.13ab	4.89±0.89ab
Σphenols	155.82±0.82f	258.8±8.15c	278.59±1.08b	325.94±0.11a	120.26±5.6g	109.13±3.51h	114.02±2.31gh	217.32±4.61e	236.33±8.31d	271.89±5.17b

Note: Results were expressed as mean ± SD (n = 3). Data of same phenolic compounds with different letters were statistically significant differences ($p < 0.05$) according to Duncan test.

Abbreviations: EGCG: (-)-Epigallocatechin gallate; GCG: (-)-Gallocatechin gallate; ECG: (-)-Epicatechin gallate.
